# Supplementary material for: Cadmium Body Burden and Inflammatory Arthritis: A Pilot Study in Patients from Lower Silesia, Poland
Source: Int J Environ Res Public Health. 2022 Mar 6;19(5):3099. doi: 10.3390/ijerph19053099 (PMC8910441; doi:10.3390/ijerph19053099)
Supplement: Supplementary file 1 [file ijerph-19-03099-s001.zip › QUESTIONNAIRE.pdf]

QUESTIONNAIRE regarding a study entitled 'Cadmium burden and inflammatory processes, antioxidant damage, and immune system regulation in individuals with autoimmune diseases'/

We would be grateful for your feedback on the following questionnaire

1. Age………………………….years

2. Sex FEMALE ☐

MALE ☐

3. Education

Higher ☐ Secondary ☐ Vocational/primary ☐

4. Occupation performed ………………………………………………..

5. Chronic diseases ………

6. Place of residence

Large city **YES NO**

Medium-sized city **YES NO**

Smaller town, village **YES NO**

Busy street, heavy car traffic **YES NO**

Periphery, residential area / low car traffic volume **YES NO**

Proximity of low emission sources / heating of homes with coal-fired furnaces **YES NO**

Exposure to passive smoking at home **YES NO**

How long have you lived in your current place of residence? …………………/ if 10 years or more → **7 points**

Previous place of residence

Large city **YES NO**

Medium-sized city **YES NO**

Smaller town, village **YES NO**

Busy street, heavy car traffic **YES NO**

Periphery, residential area / low car traffic volume **YES NO**

Proximity of low emission sources / heating of homes with coal-fired furnaces **YES NO**

Exposure to passive smoking at home **YES NO**

7. Place of work

Employment/now **YES** Employment/now **NO** (pensioner, retired, unemployed)

Job seniority ………………………….years

Place of work

Occupational exposure to cadmium / metallurgy, welding, chemical industry, plastics production, car mechanic **YES NO**

Busy street, heavy car traffic **YES NO**

Periphery / low car traffic volume **YES NO**

Proximity of low emission sources / heating of homes with coal-fired furnaces **YES NO**

Exposure to passive smoking at work **YES NO**

Commuting to work

- public transport ☐
- car ☐
- on foot ☐

How long you have been employed at your current workplace? ..../ if 10 years or more ⟶ **8 points**

Previous place of work

Occupational exposure to cadmium / metallurgy, welding, chemical industry, plastics production, car mechanic **YES NO**

Busy street, heavy car traffic **YES NO**

Periphery / low car traffic volume **YES NO**

Proximity of low emission sources / heating of homes with coal-fired furnaces **YES NO**

Exposure to passive smoking at work **YES NO**

Commuting to work

- public transport ☐
- car ☐
- on foot ☐

8. Lifestyle

Vegetarian diet **YES NO**

Frequent consumption of offal **NO** / at least once a week / **YES** several times a week / **YES**

High consumption of legumes **NO** / at least once a week / **YES** / several times a week / **YES**

Tobacco smoking **YES NO**

If **YES**, how log: ……………………………..years

Tobacco smoking **YES / addictive, about a pack a day** ☐ **half a pack a day** ☐

Tobacco smoking **YES / rarely, occasionally, e.g. at parties** ☐

Tobacco smoking **NO** ☐

Quitting smoking

How long ago? ……………………………years

How long had you been smoking before? ………………………years

Cosmetic use / for women

Lipstick **YES / every day** ☐

Lipstick **YES / occasionally** ☐

Lipstick **NO** ☐

Powder/foundation **YES / every day** ☐

Powder/foundation **YES / occasionally** ☐

Powder/foundation **NO** ☐

Eyeshadows **YES / every day** ☐

Eyeshadows **YES / occasionally** ☐

Eyeshadows **NO** ☐

Leisure time / how do you spend it most often?

At home / TV, computer, books ☐

Going out to town / cafes, cinema, events ☐

Trips out of town / excursions ☐

Walks in a park ☐

In a different way: how? …………………………………………………………………………………

Thank you for completing the questionnaire.
