# Supplementary material for: Cadmium Body Burden and Inflammatory Arthritis: A Pilot Study in Patients from Lower Silesia, Poland
Source: Int J Environ Res Public Health. 2022 Mar 6;19(5):3099. doi: 10.3390/ijerph19053099 (PMC8910441; doi:10.3390/ijerph19053099)
Supplement: Supplementary file 1 [file ijerph-19-03099-s001.zip › Equation (Binary logistic regression model).pdf]

$$P(x) = \frac{e^{\mathbf{9.9} + \mathbf{1.49} * \text{Cd–B} \geq 0.65\ \mu g/L + \mathbf{2.36} * (\text{8–OHdG} \geq 7.66\ ng/mL) + \mathbf{1.76} * \text{COX–2} \geq 22.9\ ng/mL - \mathbf{0.39} * \text{MCH} + \mathbf{1.29} * \text{frequent consumption of offal}}}{1 - e^{\mathbf{9.9} + \mathbf{1.49} * \text{Cd–B} \geq 0.65\ \mu g/L + \mathbf{2.36} * (\text{8–OHdG} \geq 7.66\ ng/mL) + \mathbf{1.76} * \text{COX–2} \geq 22.9\ ng/mL - \mathbf{0.39} * \text{MCH} + \mathbf{1.29} * \text{frequent consumption of offal}}}$$

where: P(x) – probability of IA in the study population.
